# Cancer Vaccines and Beyond: The Transformative Role of Nanotechnology in Immunotherapy

**DOI:** 10.3390/pharmaceutics17020216

**Published:** 2025-02-07

**Authors:** Violeta Delgado-Almenta, Jose L. Blaya-Cánovas, Jesús Calahorra, Araceli López-Tejada, Carmen Griñán-Lisón, Sergio Granados-Principal

**Affiliations:** 1 GENYO, Centre for Genomics and Oncological Research, Pfizer/University of Granada/Andalusian Regional Government, 18016 Granada, Spain; violeta.delgado.almenta@gmail.com (V.D.-A.); e.joselucas@go.ugr.es (J.L.B.-C.); jesus.calahorra.gm@gmail.com (J.C.); araceli.lopez@genyo.es (A.L.-T.); 2 Instituto de Investigación Biosanitaria ibs.GRANADA, 18012 Granada, Spain; 3 Department of Biochemistry and Molecular Biology II, Faculty of Pharmacy, University of Granada, Campus de Cartuja s/n, 18011 Granada, Spain; 4 Excellence Research Unit “Modeling Nature” (MNat), Centro de Investigación Biomédica (CIBM), University of Granada, 18016 Granada, Spain

**Keywords:** cancer immunotherapy, tumor antigens, nanovaccines, personalized medicine

## Abstract

Cancer is one of the leading causes of morbidity and mortality globally, responsible for approximately 10 million deaths in 2022 and an estimated 21 million new cases in 2024. Traditional cancer treatments such as surgery, radiation therapy, and chemotherapy often present limitations in efficacy and side effects. However, immunotherapeutic vaccines have emerged as a promising approach, leveraging the body’s immune system to target and eliminate cancer cells. This review examines the evolving landscape of cancer vaccines, differentiating between preventive and therapeutic strategies and highlighting the significance of tumor-specific antigens, including tumor-associated antigens (TAAs) and neoantigens. Recent advancements in vaccine technology, particularly through nanotechnology, have resulted in the development of nanovaccines, which enhance antigen stability, optimize delivery to immune cells, and promote robust immune responses. Notably, clinical data indicate that patients receiving immune checkpoint inhibitors can achieve overall survival rates of approximately 34.8 months compared to just 15.7 months for traditional therapies. Despite these advancements, challenges remain, such as the immunosuppressive tumor microenvironment and tumor heterogeneity. Emerging evidence suggests that combining nanovaccines with immunomodulators may enhance therapeutic efficacy by overcoming these obstacles. Continued research and interdisciplinary collaboration will be essential to fully exploit the promise of nanovaccines, ultimately leading to more effective and accessible treatments for cancer patients. The future of cancer immunotherapy appears increasingly hopeful as these innovative strategies pave the way for enhanced patient outcomes and an improved quality of life in oncology.

## 1. Introduction

Cancer is one of the leading causes of morbidity and mortality worldwide, responsible for approximately 10 million deaths in 2022 and an estimated 21 million new cases in 2024 [1]. It is characterized by the uncontrolled growth of abnormal cells that invade tissues and can spread to other body parts. The heterogeneity of cancer types and genetic mutations complicate accurate diagnosis and treatment [2]. Over the years, traditional treatments such as surgery, radiation therapy, and chemotherapy have been developed; however, these often present limitations in efficacy and side effects [3].

Immunotherapy has demonstrated significantly higher response rates compared to conventional treatments such as chemotherapy. For example, immune checkpoint blockade (ICB) drugs have significantly transformed cancer treatment since their initial approval in 2011, particularly for advanced melanoma. Recent data indicate that the overall survival rate for patients treated with nivolumab has reached approximately 34.8 months, compared to just 15.7 months for traditional therapies [4]. Additionally, the adoption of ICB therapies has notably increased by about 150% in clinical practice, reflecting their growing importance in the management of various solid tumors [5]. Research on cancer biology has been essential for designing more effective and specific therapeutic options, particularly in understanding the critical role of the immune system in identifying and eliminating abnormal cells, including those that have undergone malignant transformation. However, cancer cells have developed sophisticated mechanisms to evade immune detection and destruction, ensuring their survival and proliferation [6]. These mechanisms are collectively described as immune evasion and are pivotal to tumor progression and metastasis [6,7]. Immunoediting is a key process supporting immune evasion, which involves a dynamic interaction between tumor cells and the immune system. During this process, the immune system exerts selective pressure on tumor cells, eliminating those that are immunogenic while allowing less-detectable variants to proliferate. This results in a heterogeneous tumor microenvironment (TME) where cancer cells become increasingly adept at avoiding immune recognition [8]. Notably, various solid tumors, including melanoma, exhibit immune evasion mechanisms such as the high expression of PD-L1, which allows tumor cells to inhibit T-cell activation. Additionally, tumors with significant mutational burdens, such as those in non-small cell lung cancer (NSCLC), can develop resistance to immune checkpoint blockade therapies. These mechanisms are crucial to understanding how tumors can adapt and evade immune responses, highlighting the importance of developing targeted immunotherapeutic strategies [4,5].

Another critical aspect of immune evasion is the tumor’s ability to create an immunosuppressive microenvironment. Cancer cells achieve this by secreting immunosuppressive cytokines, such as transforming growth factor-beta (TGF-β) and interleukin-10 (IL-10), which inhibit the activity of effector immune cells. Additionally, tumors recruit regulatory T-cells (Tregs) and myeloid-derived suppressor cells (MDSCs) to further dampen immune responses. These actions collectively impair the function of tumor-infiltrating lymphocytes (TILs), which are essential for the immune-mediated destruction of cancer cells [6,9]. Also, a hallmark of tumor immune evasion is the downregulation or loss of tumor-specific antigens (TSAs) and major histocompatibility complex (MHC) molecules on the surface of cancer cells. This downregulation prevents effective antigen presentation to cytotoxic T lymphocytes (CTLs). Tumors may also express immune checkpoint ligands, such as programmed death-ligand 1 (PD-L1), which bind to receptors on T-cells, inhibiting their activation and promoting immune tolerance [10,11]. Due to evidence showing that the interaction between immune cells and cancer cells plays a critical role in disease prognosis, cancer immunotherapy is now considered the fourth main treatment. Despite these challenges, advances in immunology and molecular biology have provided significant insights into the mechanisms of immune evasion [7]. Emerging therapies, including immune checkpoint inhibitors, adoptive T-cell therapies, and cancer vaccines, aim to restore or enhance the immune system’s ability to recognize and eliminate cancer cells. For instance, immune checkpoint blockade therapies targeting PD-1, PD-L1, and CTLA-4 have revolutionized cancer treatment by reactivating exhausted T-cells, enabling robust antitumor responses [12,13].

In this context, the advent of vaccines has led to a distinction between preventive and therapeutic vaccines in cancer immunotherapy. Preventive vaccines aim to induce immunological memory by administering vaccines to healthy individuals, thereby preventing the morbidity associated with specific cancer types. In contrast, therapeutic vaccines are given to cancer patients to enhance or reactivate their immune responses [14]. The primary goal of cancer vaccines is to harness the immune system’s ability to eliminate tumor cells, akin to its response against infectious diseases. The integration of vaccines with immune checkpoint inhibitors, such as PD-1 and CTLA-4, has demonstrated remarkable improvements in the efficacy of these therapies. This synergy not only enhances the elimination of tumor cells but also helps overcome some of the mechanisms that tumors develop to evade the immune system [7].

Additionally, achieving an effective immune response requires meticulous antigen selection. Tumor antigens are categorized into TSAs, which are exclusive to tumor cells and often arise from somatic mutations, and tumor-associated antigens (TAAs), which may also be expressed in normal tissues but exhibit differential or aberrant expression in tumors [2,15]. An emerging area of interest is neoantigens, proteins derived from tumor-specific DNA mutations. The therapeutic potential of personalized neoantigens, derived from unique tumor mutations, has become a focal point, allowing for the development of vaccines that elicit highly specific immune responses. This personalization is key to improving efficacy and minimizing side effects [14,16]. Moreover, vaccines targeting multiple tumor antigens have been shown not only to induce specific immune responses but also to generate long-term immune memory, which is crucial for preventing relapse [17]. Research into immunotherapy has indicated that, alongside vaccines, optimizing the TME is also critical. Therapies that combine vaccines with microenvironment modulators may help reinvigorate the patient’s immune system to combat cancer more effectively [18].

**Figure 1:**
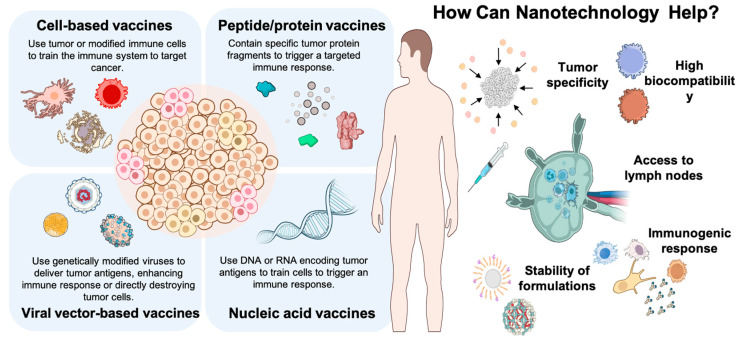
Scheme of cancer vaccine categories: employing cells, proteins, viral vectors, nucleic acids, and nanovaccines to optimize immune targeting of cancer cells through nanotechnology. The figure was created using NIAID NIH BIOART: source bioart.niaid.nih.gov (accessed on 4 February 2025).

However, cancer vaccines face unique challenges compared to those for infectious diseases. Tumors are dynamic and adaptive, capable of evolving mechanisms to evade immune surveillance [2]. In recent years, research into nanovaccines has represented a transformative advancement in cancer immunotherapy, combining nanotechnology with precision medicine to improve antigen delivery, stability, and immunogenicity (Figure 1). These nanoscale platforms ensure the targeted transport of tumor antigens and adjuvants, enhancing the immune system’s ability to identify and attack tumor cells while minimizing off-target effects. By encapsulating antigens within nanoparticles, nanovaccines protect these molecules from degradation and optimize their presentation to immune cells, thereby promoting a strong and sustained antitumor response [19,20]. One of the key advantages of nanovaccines is their capacity to co-deliver multiple tumor antigens and immune-stimulating agents, driving robust and multifaceted immune responses. Platforms such as liposomes, polymeric nanoparticles, and lipid nanoparticles have shown great potential in this regard, enabling the induction of both cellular and humoral immunity. These systems are particularly valuable for addressing tumor heterogeneity by stimulating polyfunctional T-cell responses that can target diverse cancer cell populations [21,22].

Moreover, the integration of nanotechnology into vaccine design allows for the personalization of cancer treatment by incorporating neoantigens unique to individual tumor profiles. These personalized nanovaccines promise superior specificity and reduced off-target effects, addressing the challenges of traditional immunotherapies [23]. Emerging evidence further supports the role of nanovaccines in addressing the challenges posed by immunosuppressive TMEs. The functionalization of nanoparticles with immune checkpoint inhibitors or cytokine inducers has demonstrated the improved infiltration of effector T-cells into tumors, marking a significant step toward the development of more effective cancer vaccines [24]. Clinical and preclinical studies underscore the synergy of combining nanovaccines with modulators of the tumor microenvironment. By enhancing antigen presentation and countering immune evasion mechanisms, these strategies hold promise for achieving durable antitumor immunity and reducing cancer recurrence rates [25,26].

Over recent decades, significant progress has been achieved in cancer vaccine research, driven by advances in antigen identification technologies and the development of diverse vaccine platforms. Each of these approaches has contributed to expanding the landscape of cancer immunotherapy, offering unique mechanisms for inducing antitumor immune responses. However, nanovaccines have emerged as a particularly promising innovation, providing advantages such as enhanced antigen stability, precise delivery to immune cells, and the ability to integrate multiple therapeutic agents. This review examines the mechanisms and therapeutic potential of nanovaccines, while also contextualizing them within the broader spectrum of cancer vaccine strategies, highlighting their role in shaping the future of personalized cancer immunotherapy (Figure 1).

## 2. Cell-Based Vaccines

**Figure 2:**
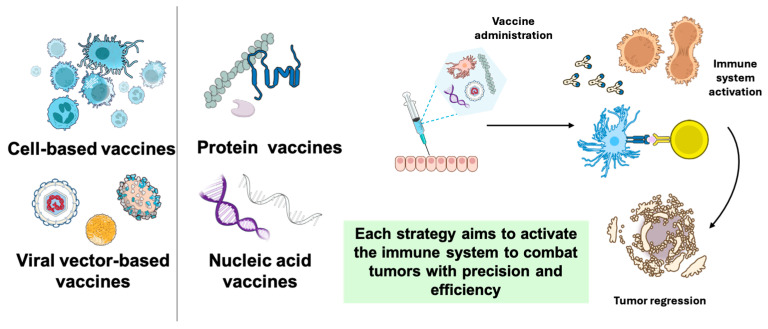
Schematic representation of major cancer vaccine strategies: cell-based, peptide, nucleic acid, and viral vector-based vaccines. The figure was created using NIAID NIH BIOART: source bioart.niaid.nih.gov (accessed on 4 February 2025).

Cell-based vaccines are prepared with either whole cells (modified or not) or part of them. These vaccines contain tumor antigens, which can boost the immune response by facilitating the identification and attack of cancer cells (Figure 2). The first vaccine based on tumor cells or tumor lysates was developed in 1980. Autologous tumor cells were used in this trial to treat colorectal cancer [27,28], and their results gave rise to greater interest from the scientific community in this type of therapy. In 2010, a dendritic cell-based vaccine (Sipuleucel-T) was successfully used to treat prostate cancer, demonstrating the feasibility and wide application of cancer vaccines [29]. We can say, then, that these type of cell-based vaccines are divided into tumor cell vaccines and immune system cell vaccines [30,31].

Tumor cell vaccines use the patient’s tumor cells (autologous) or allogeneic cell lines that contain the antigens associated with tumors and, thus, the epitopes of CD4^+^ helper T-cells and CD8^+^ CTLs. These irradiated tumor cells are administered together with an adjuvant and may be able to induce the production of specific T-cells against any antigen expressed by the tumor cells used [14,32]. However, the limiting factor is the number of cells obtained, which do not reach the necessary quantities to overcome the immunosuppressive environment of the tumor. This approach has been used in lung cancer [33], colorectal cancer [34,35], melanoma [36], and prostate cancer [37], among others. In addition, in some cases, tumor cells are genetically modified to add functions such as cytokine production (IL-21 or IL-7) [38,39] and granulocyte–macrophage colony-stimulating factor (GM-CSF) [40].

On the other hand, vaccines based on cells of the immune system make use of the role that these cells have in enhancing the immune response to cancer. This is the case with the use of dendritic cells (DCs), which are the main mediators of the presentation of antigens to the immune system [41]. These DCs are loaded with tumor antigens or lysates, and once activated, they are reintroduced to the patient to present these antigens to T lymphocytes, activating a specific immune response against the tumor [42,43]. Most DC-based vaccines are derived from monocytes, and good tolerance and efficacy results have been seen in monocyte vaccination studies [27,31,41,44]. One of the emerging strategies in this field is the use of cancer stem cells (CSCs). They are a subpopulation of the cells present in tumors that have characteristics such as self-renewal and resistance to treatment, which contribute to the development of cancer recurrence and metastasis [45,46]. Dendritic cells are sensitized to CSCs or their lysates, which could allow targeted cytotoxicity (with the greater activation of T lymphocytes against these CSCs) and decreased tumor cell migration [44,47]. There are still no FDA-approved CSC-based vaccines, but multiple in vitro trials have demonstrated their ability to inhibit tumor growth and metastasis [48,49].

### 2.1. GVAX, Provenge, and Canvaxin

GVAX is a cancer vaccine based on tumor cells modified to secrete GM-CSF. It is administered once the tumor is irradiated to stop the uncontrolled proliferation of tumor cells [50,51]. There are two modalities of the GVAX vaccine: one that uses the patient’s cells (autologous), and another that uses non-patient-specific cells (allogeneic). It has been seen in clinical trials that this vaccine has promising results in the treatment of lung carcinoma, with a relationship having been identified between GM-CSF secretion and a good prognosis of the disease [52,53]. Efficacy has also been seen in phase II clinical trials for pancreatic cancer, in combination with radiotherapy [14,54].

Sipuleucel-T is a type of vaccine based on autologous cells. It is made from a leukocyte fraction obtained from the patient’s peripheral blood, which is cultured with an antigen specific to prostate carcinoma, known as prostatic acid phosphatase (PAP), in the presence of GM-CSF [55]. DCs, which constitute approximately 11.2% of the preparation [56], are the main active components and have the function of presenting the PAP antigen to activate and induce antigen-specific T-cells in the patient. Sipuleucel-T was the first therapeutic cancer vaccine, approved for clinical use in 2010 under the name Provenge. This vaccine has been shown to increase the average overall survival by 4 months in patients diagnosed with prostate cancer [57,58,59]. Provenge is an example of a personalized vaccine, which has been created using mass spectrometry or other methods, such as exome sequencing. The objective of this type of vaccine is to identify the mutation and neoantigen characteristics of the patient’s tumors, and their great potential has been seen in clinical trials [57,60]. Finally, Canvaxin can overcome the hurdle of vaccines that use the patient’s cells, as it employs tumor cell lines. It has been studied in prostate, breast, and pancreatic cancers [14,61,62].

While all three vaccines share the goal of enhancing the host’s immune response to tumor antigens, they differ in their design and application. GVAX and Canvaxin rely on whole tumor cells, with GVAX focusing on GM-CSF secretion, while Provenge is a more targeted approach, using DCs to present a specific tumor antigen. Provenge has achieved regulatory approval with modest survival benefits, while GVAX and Canvaxin have shown promise in early trials but face challenges in demonstrating consistent efficacy in larger studies. Personalized vaccines like Provenge face logistical and cost barriers, while GVAX and Canvaxin must overcome issues related to scalability, immunogenicity, and variability in patient responses [57,58,59,60,61].

### 2.2. Improving Immunogenicity

In recent years, multiple advances have been made in the development of vaccines based on tumor cells. For this type of vaccine to be effective, it is essential to enhance their immunogenicity. These cells secrete soluble factors that could suppress immune system cells [63,64]. Some approaches would be to use dead cells instead of living cells, as these induce an enhanced immune response, modify tumor cells, or use radiotherapy [27].

To cause cell death in tumor cells, oncolytic adenoviruses, coxsackieviruses, or measles virus, among others, are often used [65]. These can produce phenotypes that result in immunogenic cell death (ICM). In addition to the host-specific damage-associated molecular patterns (DAMPs), therapy using oncolytic viruses leads to the release of pathogen-associated molecular patterns (PAMPs). This results in the increased recruitment of immune system cells into the immunosuppressive environment that tumors create to prevent their destruction [66,67]. In this way, the cross-presentation of tumor-associated antigens becomes more effective, a phenomenon by which antigen-presenting cells (APCs) capture exogenous tumor antigens and process them to present them in molecules of the main histocompatibility complex class I (MHC-I). This mechanism allows the activation of CTLs [68]. Another option is the use of oxaliplatin, but adenoviruses have been shown to induce immunogenic cell death better [69]. In the case of neuroblastoma, it has been seen that the use of nanoparticles to activate interferon genes achieves the effective apoptosis of these tumor cells [70].

When it comes to modifying tumor cells, the main objective is to improve the antigen presentation to improve the immune response. Two factors that can increase the response of T-cells are IL-21 (interleukin 21) and IL-7. Vaccines that present this genetic modification have shown great efficacy [38]. Likewise, the use of adjuvants has been shown to improve antigen presentation. As is the case in the use of nanoparticles loaded with cytosine–phosphate–guanine (CpG) to mark tumor cells in the process of apoptosis. Another way to boost immunogenicity is to use combination therapy, which involves using whole-cell vaccines along with immune checkpoint inhibitors. Blocking PD-L1, a protein that controls the body’s immune response, has already been shown to improve the response to vaccination. These immune checkpoint inhibitors work by attaching to PD-L1 and preventing it from binding to PD-1, a protein in T-cells. When this binding is impeded, T-cells become active and kill cancer cells [71,72].

When radiation therapy is used in conjunction with DC vaccines, there is a greater involvement of tumor-associated neutrophils. This means that reactive oxygen species are produced to a greater extent, which increases the damage suffered by tumor cells, leading to apoptosis. This leads to the release of tumor neoantigens, which allows DCs to mature and recognize antigens more easily. In addition, if the vaccine is used together with cytokines, chemotherapy, or adjuvants, its efficacy improves [73,74,75].

### 2.3. Limitations of Monotherapy Vaccines

Cancer vaccines in monotherapy face several limitations that restrict their clinical efficacy. This is due, among other factors, to the presence of an immunosuppressive tumor microenvironment. The tumor environment is usually dominated by immunosuppressive cells and molecules, such as regulatory T-cells (Tregs), tumor-associated macrophages (TAMs), and cytokines such as TGF-β and IL-10, which inhibit the immune response generated by the vaccine [76,77]. Likewise, a good selection of antigens to activate CD8^+^ and CD4^+^ T-cells is necessary to enhance the efficacy of our vaccine. Many tumors lack highly immunogenic neoantigens, which hinders the efficient activation of the immune system by vaccines [57]. Another key aspect is that vaccines, in monotherapy, often generate a weak or insufficient immune response to completely eradicate tumor cells, especially in the advanced stages of the disease. Also, we found limitations in terms of the type of DCs used. Monocyte-derived DCs, commonly used in vaccines, often have a limited ability to migrate to lymph nodes and efficiently activate T lymphocytes. This can reduce the generation of robust immune responses [78,79]. This could be solved by forcing them to migrate to the lymph nodes, allowing them to mature. The function of the nodules is to optimize the immune response by facilitating the interaction of APCs with T-cells, promoting their activation and proliferation. When warning signals are detected, DCs are rapidly activated, migrate from peripheral tissues to lymph nodes, and undergo a maturation process, enabling them to efficiently present antigens and trigger a robust adaptive immune response [80]. Another strategy to induce the maturation of DCs is through the use of FLT3 receptor ligands (FLT3Ls). These ligands are proteins that act as growth factors derived from the receptor tyrosine kinase type 3 (FLT3), playing a fundamental role in the differentiation, proliferation, and survival of DCs [66].

An example of this process is that of CD103+ DCs. These move from the tumor to the lymph nodes after antigen recognition occurs and are needed for cross-presentation with CD8^+^ T-cells. For this process to be carried out effectively, the CCR7 chemokine receptor is necessary [81,82]. Studies in mouse models have shown that those that are deficient in the CCR7 receptor are also deficient in CD103+ cells and that by increasing the expression of CCR7, the migration of DCs to the lymph nodes also increases [66]. It should also be taken into account that tumors undergo constant changes and mutations to escape the immune system, such as angiogenesis or the regulation of molecules with a suppressive function of the immune system [14,27]. That is why, compared to other therapeutic strategies against cancer, vaccines show limited benefits. To evaluate the efficacy of vaccines, it is also necessary to identify biomarkers in patients that allow us to see the antitumor response. Although some biomarkers, such as CXCR4 and CD32, have been proposed, their effectiveness is still limited [57], underscoring the need to develop more precise and specific indicators to assess antitumor responses in the clinical context.

### 2.4. Glioblastoma Multiforme

One of the main challenges in cancer treatment is brain tumors, particularly malignant gliomas, such as glioblastoma multiforme (GBM). This type of tumor presents multiple obstacles to be addressed by immunotherapeutic vaccines, due to [83]

- The presence of the blood–brain barrier, which limits the access of many immune system cells to brain tissue, restricting their ability to exert an effective response against tumors.
- The lack of antigens, specific to the glioma, identified.
- The ability of the glioma to create an immunosuppressive tumor microenvironment.

Since 2000, a deeper understanding of the interaction between the immune system and the central nervous system has been achieved, as well as the mechanisms that allow the selective penetration of substances through the blood–brain barrier. This advance has facilitated the identification of glioma-specific antigens, which has represented significant progress in the development of immunotherapeutic vaccines targeting this type of tumor [58,83,84]. GBM began being treated using CD14+ monocytes isolated from peripheral blood. These monocytes were differentiated into immature DCs in the presence of GM-CSF and IL-4. Antigens were then presented to the DCs, which matured in the presence of factors such as IL-4, IL-6, GM-CSF, and tumor necrosis factor α (TNF-α) [85]. The use of this type of vaccine against GBM disease was first described in 2000 by Liau et al., who managed to prolong the life of a patient by 21 months [86]. Subsequently, between 2001 and 2004 it was shown that DCs could be loaded with the peptides present on the surface of glioma cells, with good results both in terms of safety and the activation of the immune response. In that study, the overall survival of eight patients was increased by an average of two and a half years [58,87]. In a more recent clinical trial, the ICT-107 study demonstrated the importance of glioma-specific antigens in vaccine development. ICT-107 is an autologous dendritic cell vaccine pulsed with six synthetic peptide epitopes targeting glioblastoma tumor and stem cell-associated antigens: MAGE-1, HER-2, AIM-2, TRP-2, gp100, and IL13Rα2 [88]. This randomized, double-blind, placebo-controlled phase II trial evaluated the efficacy and safety of ICT-107 in newly diagnosed glioblastoma patients. Although the primary endpoint of median overall survival did not show a significant difference between the ICT-107 and control groups, progression-free survival was significantly improved in the ICT-107 cohort. Notably, patients in the HLA-A2 subgroup exhibited enhanced clinical and immunological responses to the vaccine.

It is also possible to use tumor lysates, which include a wide range of antigens associated with various types of tumors. These are presented to DCs and the MHC in the presence of cytokines such as IL-4, IL-6, TNF-α, and GM-CSF, which favors the maturation of DCs and facilitates their ability to present antigens to MHC more efficiently [89]. The disadvantage of using tumor lysates is the poor immunogenicity they may present. However, it can be solved with the presence of the cytokines mentioned above and with the use of hypochlorous acid (HOCl). The addition of GM-CSF to tumor lysate vaccines has been shown to enhance the maturation and activation of DCs, which are critical for antigen presentation. The presence of cytokines such as L-12 and IFN-γ can further promote the activation of CTLs and natural killer (NK) cells, enhancing the immune response against glioblastoma cells [90]. These cytokines also help in reshaping the tumor microenvironment, making it more conducive to immune cell infiltration and activation. HOCl has the property of enhancing the immunogenicity of tumor lysates by modifying the lysates to improve antigen presentation. This acid induces changes that increase the ability of APCs to recognize and present tumor antigens more effectively to T-cells, while also reducing the presence of immunosuppressive factors within the lysates. Additionally, it has the property of causing the cell death of the cancer cells to occur more quickly [58,91]. From a safety point of view, clinical trials of DC vaccines have been carried out in phases I and II, and hardly any adverse effects have been found, except for diarrhea, vomiting, fatigue, etc. The greatest concern about exposure to cancer antigens is the increased risk of developing cancer, but this adverse effect has not been confirmed. On the other hand, the development of autoimmune phenomena is not observed when this type of vaccination is carried out [57,92,93,94].

## 3. Peptide and Protein Vaccines

Protein- or peptide-based vaccines are designed to generate immunity against specific epitopes, which are specific regions of an antigen recognized by the immune system (Figure 2) [95]. These epitopes come from the proteins and peptides unique to cancer cells and are usually not found in normal tissues. The antigens used in these vaccines are artificially synthesized in the laboratory and, once administered, are processed by the APCs and presented on their surface in the context of MHC [96,97].

When T-cells detect these antigens, a specific immune response against the cancer is triggered. Currently, numerous tumor-associated, antigen-derived epitopes (TAAs) capable of binding to MHC have been identified [97,98]. In addition, antigens caused by mutations unique to cancer cells, known as neoantigens, have become very relevant, as they allow the immune system to attack tumor cells directly. To identify these neoantigens more efficiently, advanced computational algorithms are being developed [10,99].

Although the early phases of clinical trials with protein and peptide vaccines have shown encouraging results, most phase III trials have not achieved the expected success [10,100]. This is mainly because the antitumor responses generated have not been effective enough. Even when T-cell responses were activated, the success rates in terms of tumor clearance were low, with less than 10% efficacy in some cases [101]. This limited result can be explained by factors such as the immune escape mechanisms of the tumor and the immunosuppressive environment that forms in the tumor microenvironment [14].

### 3.1. Classification and Clinical Trials of Peptide Vaccines

To ensure the efficacy of a peptide-based vaccine, it is critical to select antigens that are specifically expressed on the surface of tumor cells and that are recognized by T-cells. Additional key factors include the optimal length of peptide chains, the diversity and quantity of the peptides used, as well as the strategic choice of targeted epitopes. In the latter aspect, it is crucial to decide whether to prioritize the epitopes capable of activating CD4^+^ or CD8^+^ T-cells [102].

Peptide vaccines are mainly divided into two categories: long-chain peptide vaccines and short-chain peptide vaccines [103,104]. Long-chain formulations have the advantage of being processed and presented by both MHC class I and class II molecules. This ability allows APCs to activate both CTLs and helper T-cells, thus optimizing the efficacy of the CTL-mediated antitumor response. More importantly, the processing of these long-chain peptides prevents the induction of anergy in T-cells, thanks to the simultaneous activation of specific T-cell receptors and costimulatory molecules, which ensures robust immune signaling [2,105,106]. Currently, efforts in the development of these vaccines are focused on including epitopes capable of effectively stimulating both CTLs and helper T-cells, maximizing the synergy between both cell populations in the context of immunotherapy [107]. Most of the peptide vaccines developed to date are based on short-chain peptides, which are presented exclusively by MHC class I molecules [108]. These peptides can bind directly to cells without requiring prior intracellular processing. However, when these peptides are presented to CD8^+^ T-cells in the absence of adequate costimulatory signals, immune tolerance can be induced, promoting an environment conducive to the development and progression of cancer [109]. These types of vaccines have been investigated in several types of cancer. In 2017, the FDA approved the use of DSP-7888, an innovative peptide vaccine designed to treat Wilms tumor, a rare type of kidney cancer [110]. On the other hand, widely used vaccines such as Cervarix, Gardasil, and Gardasil 9 also belong to the category of peptide vaccines and have been approved for therapeutic use. These vaccines target human papillomavirus (HPV), an etiologic agent linked to cervical and anal cancer [110,111]. However, despite advances, peptide vaccines have shown limited clinical results in terms of therapeutic efficacy in clinical trials, highlighting the need to optimize their design and approach [112].

Currently, peptide-based vaccines are being evaluated in clinical trials for various types of cancer, including lung cancer [113], pancreatic cancer [108], and melanoma [114]. Mittendorf et al. demonstrated in clinical studies that E75, a peptide derived from human epidermal growth factor receptor 2 (HER2), is safe and effective, advancing to phase III clinical trials [115]. In addition, phase II research suggested that the combination of GP2 and GM-CSF could prevent relapses in breast cancer patients. This peptide vaccine was also found to show a particularly promising performance in patients with HER2-positive tumors who had been previously treated with trastuzumab, suggesting a synergistic approach to reduce the risk of recurrence in this specific subgroup of patients [106].

Survivin is a protein with anti-apoptotic functions that is widely expressed in various types of cancer. A vaccine developed with this antigen incorporated three short-chain peptides containing multiple epitopes: eight specific to CD4^+^ T-cells and six to CD8^+^ T-cells [116]. This design allowed for the simultaneous activation of both T-cell subtypes, promoting a robust immune response. In preclinical studies, this vaccine was evaluated in murine models of colorectal cancer and B lymphoma. The results showed remarkable efficacy in inhibiting tumor growth, in addition to significantly extending the survival of treated mice, achieving an increase of up to 60 days in life expectancy [117].

Mucin-1 (MUC-1) is a glycoprotein that has a nucleus formed by a sequence of 20 amino acids repeated in tandem, known as variable number tandem repeats (VNTRs). In the context of cancer, this protein exhibits aberrant glycosylation with a reduction in carbohydrate side chains [118,119]. This structural change exposes more immunogenic epitopes of MUC-1, which activates both the antibody response and the CTL response. In a clinical trial, the injection of a MUC-1-derived peptide containing five VNTR repeats was evaluated in nine breast cancer patients. The results showed that, in seven of them, there was a significant increase in the production of IgG and IgM immunoglobulins specific to the MUC-1 peptide [120].

Finally, the Ras oncogene is part of one of the main cell signaling pathways, known as the Ras/MAPK pathway, which regulates processes such as cell proliferation, differentiation, and survival [121]. Under normal conditions, Ras remains inactive in the cytoplasm of the cell. Its activation occurs when a ligand, such as a growth factor (e.g., EGF, epidermal growth factor), binds to its specific receptor on the surface of the cell, usually a receptor tyrosine kinase (RTK). However, in many cases of pancreatic and colorectal cancer, Ras exhibits constitutive activation due to mutations, which causes uncontrolled cell division and contributes to tumor growth [122,123]. Several clinical trials have been conducted focused on immunizing against the Ras protein, using adjuvants and peptides of various lengths designed to bind to APCs. Although these studies failed to demonstrate significant clinical responses in terms of tumor shrinkage, they did show a positive correlation between immune cytokine activation and a modest increase in patient survival [120].

### 3.2. Improving the Efficacy of Peptide Vaccines

To increase the efficacy of peptide-based vaccines, a strategic selection of the peptides used is critical. This can be achieved by using computational algorithms designed to analyze the interaction between peptides and MHC molecules. These algorithms leverage detailed information about the amino acid sequence and the three-dimensional structure of the peptides, allowing their binding affinity to MHC to be accurately predicted [124]. In addition, whole-exome sequencing techniques offer a complementary approach by identifying tumor cell-specific neoantigens. These antigens, generated by unique somatic mutations of the tumor, are not present in normal tissues, which makes them ideal for designing highly immunogenic vaccines with a lower risk of inducing autoimmune responses [112,125]. In this context, personalized vaccines targeting neoantigens have demonstrated significantly higher success rates. These vaccines are designed and synthesized based on the unique genetic and molecular characteristics of each patient’s tumors [126]. The integration of the techniques with select neoantigens with a high affinity for MHC has made it possible to predict the efficacy of these vaccines more accurately, optimizing the antitumor immune response and improving therapeutic results.

One of the main challenges of peptide vaccines is their limited ability to present antigens effectively. This problem can be addressed using TLR adjuvants or agonists, which enhance immune signaling and increase the effectiveness of the immune response [127,128]. Adjuvants work by stimulating the immune system to enhance T-cell activation and antigen presentation, thereby optimizing the response against the vaccine antigen. Among the most used adjuvants are alum, monophosphorylated lipid A, polyinosinic-polycytidylic acid, CpG oligodeoxynucleotides, and the emulsifier Montanide ISA 51 [129,130,131,132,133]. In addition, GM-CSF is also employed as an adjuvant due to its ability to recruit and activate antigen-presenting cells [106,112]. Alum is present in many vaccines that are already authorized for therapeutic use by the FDA. Its main function is to enhance the immune response by facilitating the activation of APCs. On the other hand, monophosphorylated lipid A acts as a Toll 4 receptor (TLR4) agonist, promoting the activation of DCs and improving the stimulation of CTLs. Similarly, CpG oligodeoxynucleotides interact with TLR9, while polyinosinic–polycytidylic acid acts on TLR3. Both agonists stimulate DCs, increase antigen presentation, and amplify immune activation. Clinical studies have shown that the incorporation of these adjuvants into peptide-based vaccines significantly improves their efficacy [106,131]. Montanide ISA 51 works by enhancing both the immune response and the extended-release effect of vaccines [134]. This adjuvant remains at the injection site for weeks or even months, ensuring the continued presence of the epitopes capable of activating T-cells. In this way, the immune response is optimized, which improves the effectiveness of vaccines. Several studies have shown that the combination of Montanide with the peptides of the oncoproteins E6 and E7 stimulates both CD4^+^ and CD8^+^ T lymphocytes, favoring a more robust and tumor-directed immune response [106,133]. The last adjuvant to mention is GM-CSF. Its mechanism of action consists of attracting DCs to the skin, where the tumor epitopes are found. In addition, it has been observed that GM-CSF can contribute to antiangiogenesis, which limits the blood supply to tumors, preventing their growth. This adjuvant has been used in the final stages of clinical trials. As for the adverse effects associated with GM-CSF, the most common and serious detected has been a local reaction at the injection site [106,135].

### 3.3. Combination with Other Therapies

One of the main drawbacks of peptide-based vaccines is their limited efficacy when given as monotherapy. However, their therapeutic performance is significantly improved when combined with other antitumor strategies or immunomodulatory drugs. A prominent example of an effective combination is the co-administration of peptide vaccines with immune checkpoint inhibitors [117]. Immune checkpoint inhibitors regulate the response of T-cells by blocking molecules that act as brakes on immune activation, such as PD-1/PD-L1 and CTLA-4. These molecules normally prevent autoimmune responses by limiting T-cell activity [136]. However, in the tumor context, they can also reduce the function of CTLs, allowing cancer cells to escape immune control. Blocking these checkpoints with inhibitors ensures that CTLs maintain their ability to specifically target tumor cells [137].

In addition, peptide vaccines can be combined with other existing antitumor therapies. We have previously mentioned the anti-HER2 monoclonal antibody, trastuzumab, used in the treatment of breast cancer. Trastuzumab facilitates the elimination of tumor cells by T-cells and antibodies, increasing the vulnerability of malignant cells [138]. In one study, anti-HER2 antibodies were shown to enhance the response of DCs and specific CTLs to HER2-derived peptides, compared to using the peptide alone. In a phase II clinical trial, the combination of trastuzumab with GM-CSF and E75 peptide demonstrated that the vaccine was safe and had no significant adverse effects [117,139,140].

An additional combination under investigation is the use of cyclophosphamide in conjunction with personalized vaccines. Cyclophosphamide, at high doses, exerts cytotoxic effects, while at low doses it acts as an immunomodulator [141,142]. A phase II clinical trial evaluated the efficacy of personalized vaccines in combination with cyclophosphamide in patients with biliary tract cancer. Although no significant differences were observed in therapeutic efficacy compared to the use of the vaccine alone, there was evidence of an increase in survival in patients treated with the combination. This benefit was attributed to the reduction in the levels of interleukin-6 (IL-6), a cytokine associated with a better clinical prognosis [143].

## 4. Nucleic Acid Vaccines

Nucleic acid vaccines, encompassing DNA- and RNA-based platforms, represent a groundbreaking approach to cancer immunotherapy. By delivering genetic material into hosT-cells, these vaccines enable the production of TAAs, which activate the immune responses capable of targeting and destroying cancer cells (Figure 2) [144,145]. They are also quite stable, safe, and effective [14,146]. DNA-based immunization began in the 1990s with the discovery of a plasmid DNA encoding the nucleoprotein of the influenza A virus. DNA vaccines need to reach the cytoplasm of APCs to access the nucleus and initiate transcription, a crucial step in the process. However, RNA vaccines do not integrate into the genome, which prevents carcinogenic effects. Additionally, RNA vaccines do not need to enter the nucleus, resulting in a very low likelihood of adverse effects. Their only disadvantage compared to DNA vaccines is that they degrade more easily, but their stability can be increased by using liposome formulations or stabilizing adjuvants [146,147]. Their versatility allows for personalization and the tailoring of vaccines to individual tumor profiles. Recent advancements in delivery systems, such as lipid nanoparticles and biodegradable polymers, have improved their efficacy and safety, paving the way for next-generation, patient-specific treatments. Both platforms hold promise but require advancements in delivery systems and safety optimization to fully realize their therapeutic potential [145,148].

### 4.1. DNA Vaccines

DNA vaccines have emerged as a promising approach in cancer immunotherapy due to their ability to induce strong immune responses by delivering genetic material that encodes tumor antigens. These vaccines can be plasmid-based or mRNA-based, with each offering distinct advantages. Plasmid DNA vaccines are advantageous because they are stable, easy to produce, and can be designed to encode specific antigens, allowing for targeted immune activation. They stimulate both cellular and humoral immunity, making them effective in inducing robust immune responses against cancer cells [148,149]. Recent research has shown that the combination of DNA vaccines with adjuvants or the use of novel delivery systems, such as nanoparticles, can significantly enhance vaccine effectiveness by improving antigen presentation and boosting immune responses. Moreover, DNA vaccines targeting tumor-specific neoantigens are gaining traction, offering the potential for personalized cancer immunotherapy. These vaccines can be tailored to the individual’s tumor profile, which may increase specificity and reduce off-target effects [150].

Strategies to enhance DNA vaccines for cancer focus on improving immune responses by optimizing antigen delivery, immune cell activation, and overcoming tumor-induced immune suppression. One promising approach is the “prime-boost” strategy, which combines an initial DNA vaccine administration with subsequent vaccination using viral or bacterial vectors encoding the same antigen. This strategy increases the production of antigen-specific T-cells and activates TLRs, stimulated by danger signals from bacterial vectors. This method has shown positive results in hepatocellular carcinoma patients. Clinical trials are also investigating a vaccine encoding HER-2 and carcinoembryonic antigen (CEA), combined with an *E. coli* toxin [151]. Another effective approach is combining DNA vaccines with immunomodulators such as cytokines. Cytokines stimulate immune responses, and their co-administration with DNA vaccines using plasmids encoding cytokines such as IL-2 and GM-CSF enhances T-cell activation and dendritic cell maturation without causing systemic toxicity. IL-2, approved for use in metastatic melanoma and renal carcinoma, promotes T-cell maturation and activation. GM-CSF, often used in clinical trials, plays a role in dendritic cell maturation and T-cell activation by promoting the production of granulocytes and macrophages that assist in antigen presentation [147]. On the other hand, Chimeric DNA vaccines, which combine elements from xenogeneic species with self-antigens, have shown promise by stimulating a robust immune response without tolerance issues. However, studies suggest that self-antigens generally produce a stronger and more specific antibody response. To enhance this effect, hybrid plasmids combining xenogeneic and autologous antigens, such as the plasmid encoding the neu-HER2 antigen for targeting ErbB2+ tumors, are being developed [152].

Furthermore, DNA nanovaccines have gained significant attention in cancer immunotherapy due to their ability to enhance the immune response by combining the benefits of DNA vaccination with the targeted delivery capabilities of nanomaterials. By encapsulating tumor-associated antigens within nanoparticles, these vaccines not only improve the stability and efficiency of antigen delivery but also allow for the precise targeting of immune cells, reducing potential side effects [153,154]. Various nanomaterials, including liposomes, dendrimers, and polymeric nanoparticles, have been explored as carriers for DNA vaccines, offering improved penetration into tumor tissues and the better activation of immune responses. In addition, nanoparticle-based systems can incorporate immune modulators or adjuvants, further boosting the vaccine’s effectiveness by enhancing the body’s immune recognition of cancer cells [154,155]. These advancements are leading to more effective and personalized cancer immunotherapies, overcoming previous limitations in DNA vaccine delivery and providing new hope for more robust cancer treatments.

### 4.2. Messenger RNA (mRNA) Vaccines

mRNA vaccines have gained significant attention as a promising cancer immunotherapy due to their ability to induce both humoral and cellular immune responses. These vaccines deliver synthetic mRNA encoding tumor-associated antigens, which are translated into proteins by host cells. These proteins are then presented to MHC molecules, activating CD8^+^ and CD4^+^ T-cells, essential for an effective antitumor response. A notable advantage of mRNA vaccines is their rapid production and cost-effectiveness, as they can be synthesized quickly without cell-based systems. Additionally, mRNA vaccines do not integrate into the host genome, eliminating concerns about insertional mutagenesis and reducing the risk of toxicity [156,157]. Recent studies have highlighted the importance of personalized cancer vaccines, where mRNA vaccines are designed to target neoantigens unique to an individual’s tumor, further improving the specificity and efficacy of the treatment [158]. In addition to neoantigens, mRNA vaccines can be combined with other immunotherapies, such as immune checkpoint inhibitors, to enhance the overall antitumor response. These combination therapies hold great potential for overcoming the resistance mechanisms that tumors often develop against single-agent treatments [159]. One of the major advantages of mRNA vaccines is their rapid production and cost-effectiveness, as the mRNA can be synthesized quickly without the need for cell-based systems. To improve the efficacy of mRNA vaccines, researchers focus on optimizing their formulation. Key modifications, such as adding a 5’ cap structure and a Poly(A) tail, enhance the stability of the mRNA, which is crucial for its successful translation and immune activation [160].

To enhance their efficacy, adjuvants and delivery systems play a pivotal role. Lipid nanoparticles (LNPs) protect mRNA from degradation and improve cellular uptake, facilitating efficient antigen presentation. However, challenges like inflammatory responses to LNPs underscore the need for alternative systems. Emerging approaches, such as polymeric micelles, offer promising solutions by ensuring stability and minimizing adverse effects while still enhancing immune activation [157,160]. LNPs, comprising cholesterol, ionizable lipids, and polyethylene glycol (PEG), are effective at promoting the cytoplasmic delivery of mRNA by facilitating endosomal escape, a crucial step for successful antigen translation [161]. Moreover, LNPs are commonly used to deliver the mRNA into cells, protecting it from degradation and facilitating its uptake by APCs [162]. Additionally, hybrid and biomimetic systems have been developed to incorporate targeting features that minimize side effects while maximizing therapeutic specificity [163]. Research also emphasizes the functionalization of nanoparticles to modulate immune responses, optimizing both antigen presentation and T-cell activation. For instance, nanoparticles with polymeric coatings have demonstrated an enhanced ability to elicit robust adaptive immune responses [164]. Emerging technologies further explore the use of polymeric micelles and next-generation carriers, which significantly improve the stability and bioavailability of mRNA while reducing adverse effects compared to traditional delivery systems [165].

Ongoing clinical trials and preclinical studies continue to explore the full potential of mRNA-based vaccines in treating cancer, with promising results suggesting that mRNA vaccines could play a key role in the future of cancer immunotherapy [166].

### 4.3. Clinical Outcomes in DNA and RNA Cancer Vaccines

Clinical trials for DNA vaccines in cancer therapy have shown promising results. VGX-3100, targeting HPV-related cervical cancer, demonstrated safety and improved CD8^+^ T-cell responses, leading to ongoing phase III trials. GX-188E, another HPV vaccine, showed success in combination with pembrolizumab for advanced cases, enhancing immune activity against viral proteins. In breast cancer, a mammaglobin-A DNA vaccine boosted immune responses and survival without significant side effects, showing potential alongside chemotherapy and endocrine therapy [147,151]. For instance, a phase I trial evaluated a DNA vaccine targeting the intracellular domain of ERBB2 (HER2) in patients with advanced HER2-positive breast cancer. The study confirmed the vaccine’s safety and identified the optimal dose (100 μg), which elicited robust ERBB2-specific T-cell responses. These immune effects suggest potential synergy with other therapies, paving the way for combination strategies to improve clinical outcomes [167]. Another clinical trial produced data related to the personalized neoantigen vaccine NEO-PV-01 in combination with standard-of-care chemotherapy and anti-PD-1 therapy for the first-line treatment of advanced non-squamous non-small cell lung cancer (NSCLC). A phase Ib clinical trial (NCT03380871) demonstrated the feasibility and safety of this regimen. The trial showed promising antitumor activity with a high objective response rate and improved progression-free and overall survival, particularly among patients who completed the vaccination regimen [168]. In glioblastoma patients, a phase I/Ib clinical trial explored personalized neoantigen vaccines in newly diagnosed patients. The vaccine was safe and feasible, generating robust neoantigen-specific T-cell responses in dexamethasone-free patients, with evidence of these cells infiltrating the tumor. While all the patients eventually experienced disease progression, the observed median progression-free and overall survival times (7.6 and 16.8 months, respectively) suggest the potential clinical benefit, warranting further investigation in larger, controlled trials [169]. Lastly, another study’s AMPLIFY-201 phase I trial evaluated ELI-002 2P, a novel lymph-node targeted vaccine, in patients with minimal residual KRAS-mutated pancreatic or colorectal cancer. ELI-002 2P was well-tolerated, eliciting robust, multi-epitope-specific T-cell responses in 84% of patients, strongly correlating with tumor biomarker reduction (84%) and improved relapse-free survival. The vaccine’s amphiphilic design promotes lymph node delivery and robust T-cell activation [170].

Regarding mRNA vaccines, a phase I trial (NCT04161755) assessed autogene cevumeran, a personalized RNA neoantigen vaccine, in resected pancreatic ductal adenocarcinoma (PDAC) patients. The vaccine was safe and readily administered post-surgery. It induced robust, polyfunctional neoantigen-specific CD8^+^ T-cell responses in half of the patients, correlating with significantly longer recurrence-free survival (RFS) in a biomarker-evaluable cohort. A CloneTrack analysis revealed vaccine-expanded T-cell clones that persisted long-term and re-expanded upon boosting [171]. Preclinical research using a murine model of metastatic pancreatic ductal adenocarcinoma (PDAC) revealed that combining anti-CD137 agonist antibody with the GVAX vaccine and an anti-PD-1 blockade significantly improved survival, increasing the costimulatory molecule expression of tumor-infiltrating T-cells and boosting activated effector memory T-cells within the tumor microenvironment. This finding, supported by a correlation between high CD137 expression and increased CD8^+^ T-cell density in human PDAC samples, led to a clinical trial (NCT02451982) incorporating this combination therapy [172]. Another example of advancement in the clinic with these vaccines is the phase I/II clinical trial (NCT03480152) that tested mRNA-4650, a novel mRNA vaccine incorporating multiple neoantigens, predicted neoepitopes, and driver gene mutations, in four patients with metastatic gastrointestinal cancer. The vaccine was safe and generated neoantigen-specific T-cell responses, including the identification of KRASG12D-specific T-cell receptors. However, no objective clinical benefit was observed. Further research combining mRNA-4650 with checkpoint inhibitors or adoptive T-cell therapy is warranted [173]. Despite promising results, clinical trials of DNA and RNA vaccines face several limitations, particularly relating to immune escape mechanisms. Tumors and certain pathogens can develop strategies to evade the immune response, such as losing specific antigen expression or creating an immunosuppressive microenvironment, which may reduce the effectiveness of vaccines [174]. To overcome these limitations, the combination of vaccines with other therapies has been explored. For example, immune checkpoint inhibitors, such as anti-PD-1 or anti-CTLA-4, can enhance the immune response by removing the regulatory brakes on the immune system, allowing for a more effective action against tumor or infectious cells [175]. Additionally, combining vaccines with chemotherapy or radiotherapy may modify the tumor microenvironment, making it more conducive to an effective immune response. These combined strategies aim not only to improve the initial immune response but also to maintain it over the long term, reducing the chances of immune escape and increasing progression-free and overall survival in patients [176].

## 5. Viral Vector-Based Vaccines

In recent decades, viruses have aroused a growing interest in the field of cancer immunotherapy due to their ability to interact in a specific way with the immune system and the tumor microenvironment. Their ability to infect host cells, integrate into the genome, and replicate gives them unique properties. These characteristics allow viruses to act as vectors for gene transfer, as adjuvants that enhance the immune response, or even as oncolytic agents capable of destroying tumor cells directly (Figure 2) [177,178]. As has been mentioned, achieving robust cellular immunity is one of the key requirements for cancer vaccines to be effective. This can be achieved using viral vectors, which not only facilitate the efficient expression of specific antigens but also stimulate the immune system by boosting T-cell activity [179,180]. Among the most commonly used vectors are adenovirus [181], poxvirus [182], and vaccinia virus [183]. Adenoviruses have been utilized to deliver tumor antigens into muscle tissue, leveraging their capacity for efficient transfection to stimulate a robust immune response. Modified Vaccinia Ankara (MVA), a genetically engineered vaccinia virus, has shown promise in targeting renal cell carcinomas and epithelial tumors by addressing the overexpression of the MUC1 antigen [184]. Furthermore, the therapeutic vaccine PROSTVAC, which employs two poxvirus vectors to express a prostate-specific antigen, has demonstrated notable efficacy when combined with immune checkpoint inhibitors. Clinical trials revealed that PROSTVAC extended overall survival by 8.5 months and reduced the mortality risk by 44%, highlighting its potential in advanced prostate cancer therapy [14,185,186].

### 5.1. New Approaches in Vaccines Based on Viral Vectors: Heterologous Prime-Boost Vaccination

Heterologous prime-boost vaccination technology represents an innovative immunization strategy that outperforms traditional forms of vaccine administration. Unlike conventional methodologies, where the same vaccine with the same antigens is repeatedly used, this approach combines different types of vaccines administered in specific sequences [187]. In a model of Mycobacterium bovis, heterologous prime-boost vaccination with the bacillus Calmette–Guérin vaccine and DNA encoding Hsp65, Hsp70, and Apa was shown to protect against bovine tuberculosis, regardless of the order of administration [188]. However, in other models, the order proved to be crucial. In a study with a murine antigen of HSV-2 gD, starting with DNA (prime) was found to be essential, as reversing the sequence with a booster protein resulted in antibody levels comparable to a homologous protein–protein vaccination, but without improvements in helper T-cell activation [189]. In the case of a hepatitis C antigen, a boost with adenoviral vectors after a prime with DNA generated the highest levels of Th1-type CD4^+^ T-cells, outperforming both the homologous approach and other combinations of DNA and viral vectors [190].

A primate study conducted by the University of Washington compared the antibody response using vaccinia virus vectors and DNA as the “prime,” concluding that these combinations were superior to the repeated use of DNA or viral vector vaccines. This approach has also shown potential in cancer immunotherapy [187]. For example, vaccination with a prostate antigen 6-transmembrane epithelial (STEAP) combined with heterologous DNA and replicon particles similar to the Venezuelan equine encephalitis virus significantly improved the immune response, increasing the levels of IFN-γ, TNF-α, and IL-12, and delayed the tumor development in murine models [191]. This type of vaccine allows administration through different routes, adapting to specific needs. Although most studies have used conventional routes, such as subcutaneous or intradermal, recent research has shown that intratumoral vaccination is safe and effective. An example is the rF-CEA-TRICOM vaccine, which was preclinically evaluated by intratumoral administration in carcinoembryonic antigen (CEA)-positive tumors [192]. In these studies, this strategy was combined with an initial subcutaneous (prime) vaccination with rV-CEA-TRICOM, followed by a booster with rF-CEA-TRICOM. These trials have been the basis for moving towards a clinical trial in patients with prostate cancer, where this strategy has been shown to be both safe and feasible [186].

### 5.2. Types of Viral Vectors

#### 5.2.1. Adenovirus Vectors

Several viral vectors have been evaluated in clinical trials as vaccination tools directed against tumor-associated antigens. Among them, adenoviruses stand out for being non-replicative, not causing disease in humans, and possessing a wide tissue tropism. Non-human primate adenoviruses (NHPAds), such as those derived from gorillas and chimpanzees, have shown a superior immune response, especially in the activation of CD8^+^ T-cells. This is attributed to their low seroprevalence in the human population, which minimizes interference from pre-existing antibodies [179,193]. An additional advantage of NHPAds is their ability to host large genetic inserts, allowing the expression of numerous neoantigens. This feature is crucial for addressing tumor heterogeneity and preventing immunoediting-mediated immune escape. Vectors can express antigens spanning up to 2200 amino acids, which is equivalent to more than 80 neoantigens of 27 amino acids each [194]. However, a significant challenge of these vaccines is the development of anti-vector immunity after the first immunization, which may limit their effectiveness following subsequent doses [195]. To overcome this limitation, it has been shown in clinical trials that the heterologous prime-boost vaccination strategy, which uses different vector platforms, generates a more robust and broader immune response compared to protocols that repeatedly employ the same type of viral vector [179].

#### 5.2.2. Poxvirus Vectors

Poxviruses are double-stranded DNA viruses with a linear genome that stand out as widely used vectors in gene transfer, thanks to their history in vaccination programs. They are classified into two subfamilies: *Chordopoxvirinae* and *Entomopoxvirinae*. These viruses can host large DNA inserts, and attenuated strains such as MVA have demonstrated the stable and efficient expression of recombinant antigens. Vaccinia virus, belonging to the genus Orthopoxvirus within the subfamily *Chordopoxvirinae*, is especially relevant in this context [15,196]. MVA has been used in more than 120,000 individuals in smallpox eradication programs, showing an excellent safety profile in multiple clinical and preclinical trials. These characteristics position it as a promising vector in the development of cancer vaccines, particularly in heterologous prime-boost strategies [197]. Its advantages include a fast life cycle that allows viral production in 6 h, three different mechanisms of propagation that ensure its efficient dissemination, the ability to incorporate large DNA sequences to express multiple antigens, its safety in not causing disease in healthy humans, and the extensive clinical knowledge accumulated from its historical use in the smallpox vaccine [196,198].

### 5.3. Clinical Advances in Viral Vector Cancer Vaccines

MVA-based vaccines are being developed and evaluated for various applications in cancer immunotherapy, showing promising results in different indications. Clinical trials have shown that recombinant MVA, given alone or in combination with chemotherapy, induces a potent immune response [186,199]. For example, the company Transgene is researching an MVA that expresses MUC-1 and IL-2 to treat lung and prostate cancers. In patients with advanced-stage lung cancer (stages III and IV), the combination of this vaccine with cisplatin or vinorelbine has shown high efficacy, achieving disease control in 71% of patients in a phase II clinical trial. It should be noted that most of these patients had stage IV cancer with tumors positive for MUC-1, a marker of poor prognosis [200,201,202]. In addition, Transgene has developed another recombinant MVA for the treatment of cervical cancer, designed to express the E6 and E7 viral antigens of human papillomavirus type 16, along with IL-2. This approach seeks to generate a cellular response capable of eliminating both the damage caused by cancer and precancerous lesions. In three phase I studies, this vaccine has demonstrated an adequate safety profile, paving the way for more advanced research [203]. On the other hand, Oxford Biomedica is developing the TroVax vaccine, an MVA that encodes the oncofetal antigen 5T4, present in most epithelial cancers, including colon, kidney, breast, and ovary. Initial studies have evidenced the stable control of the disease, along with consistent antibody and T-cell responses, supporting its clinical potential. TroVax has also been evaluated in combination with the FOLFOX chemotherapy regimen in a phase II clinical trial, in which all 23 patients in the study developed cellular and/or humoral responses against the 5T4 antigen [199,204].

Avipoxviruses have been extensively investigated in animal models and clinical trials. ALVAC, an attenuated canarypoxvirus, has demonstrated its ability to induce significant levels of CD8^+^ T-cells in mammals, being particularly promising in the treatment of melanoma [199]. In one study, the ALVAC-GP-100 vaccine, combined with a GP-100 peptide booster, was administered to patients with metastatic melanoma, achieving a response in 8 of the 18 treated patients [205]. In addition, a phase I clinical trial evaluated the safety and efficacy of the ALVAC-CEA-B7.1 vaccine in patients with advanced adenocarcinomas expressing CEA. Increasing doses were administered by intramuscular injection every four weeks for three months. In three patients, the clinical stability of the disease was observed, correlated with an increase in CEA-specific precursor T-cells. Specific immune responses were increased with repeated vaccinations [206].

Published research on the application of adenoviruses in cancer immunotherapy remains limited. Phase I clinical trials included 54 patients who received increasing doses of a recombinant adenovirus designed to encode the melanoma antigens MART-1 or gp100, either individually or in combination with IL-2. However, immunological evaluations did not reveal consistent responses [207]. In another phase I study, a recombinant adenovirus expressing the L523S tumor antigen was used to enhance a plasmid DNA vaccine that also encoded this antigen. Although almost all patients showed a significant increase in anti-adenovirus antibody levels, only one of the ten evaluable patients developed specific antibodies against L523S. Two patients experienced relapses of the disease, while the others survived for a follow-up of 290 days [208]. These studies underscore the safety of the approach but also show the limited activation of the immune system. This can be explained by the high seroprevalence and high levels of pre-existing immunity against adenoviruses in the population. In preclinical studies and early clinical trials, it has been shown that high levels of antibodies against adenovirus can reduce the efficiency of gene transfer and decrease the effectiveness of adenovirus-based vaccines [199].

## 6. Nanovaccines

Despite important advances in cancer vaccination systems, challenges such as rapid antigen degradation, the low stability of formulations, and limited immunogenicity persist, making these strategies less effective. In this context, recent developments in nanotechnology offer a promising solution. This discipline allows for the design of more stable and targeted delivery systems, capable of protecting antigens from degradation and improving their presentation to immune cells, thus increasing the efficacy and specificity of immune responses [209,210,211]. Nanovaccines use a variety of materials to enhance the antitumor response. Among the most widely used are CpG oligodeoxynucleotides and fluorinated polyethylenimines, which activate TLRs, promoting a robust adaptive immune response. Polymers, lipid nanoparticles, and endogenous transporters are also employed [212], which improves antigen stability and specificity. Additionally, the activation of the interferon gene stimulator (STING) pathway induces the high production of type I interferons, which has prompted the use of stimulants of this pathway in the design of nanovaccines for a more efficient immune response [213]. Notwithstanding advances in cancer vaccination systems, challenges such as rapid antigen degradation, the low stability of formulations, and limited immunogenicity remain. Recent developments in nanotechnology offer promising solutions for enhancing the stability and targeted delivery of antigens, potentially improving immune responses [214]. However, scalability remains a significant concern, especially for complex systems like biomimetic and hybrid nanoparticles. Producing these nanovaccines consistently at scale requires careful optimization to ensure quality and efficacy [215]. Additionally, stability during storage is critical, as sensitive components may degrade, necessitating the development of more robust formulations [216]. Regulatory hurdles also pose challenges, as extensive safety and efficacy data are required before approval for clinical use, impacting accessibility for broader applications [217].

Nanovaccines offer multiple advantages compared to traditional cancer vaccines. First, the use of polymers allows vaccines to be activated specifically in the tumor microenvironment, facilitating the arrival of the antigen to APCs [218]. In addition, lipid nanoparticles increase the stability of formulations, simplifying large-scale production. At the same time, since the use of exogenous materials can elicit unwanted responses in other tissues, biomimetic nanovaccines based on cell membranes derived from autologous materials have been developed. These nanovaccines have shown potential in the field of effective cancer immunotherapy [219,220,221]. In addition, the small size of nanobiomaterials (1–200 nm) allows efficient access to lymph nodes, which contain the APCs essential for the cross-presentation of antigens and the activation of CD4^+^ T-cells [222,223]. The cross-presentation of antigens is performed through two main pathways: the vacuolar pathway and the endosome–cytosol pathway. In the vacuolar pathway, the antigens are processed within lysosomes and loaded into MHC-I for presentation to the T-cells. In the endosome–cytosol pathway, the antigens are transported to the cytosol, where they are degraded by the proteasome; the generated peptides are transferred to the endoplasmic reticulum to be loaded into MHC-I. This mechanism improves the immunological efficacy of nanovaccines against tumors, overcoming the limitations of traditional strategies [209].

### 6.1. Efficiency of Nanovaccines in Activating the Immune System

**Figure 3:**
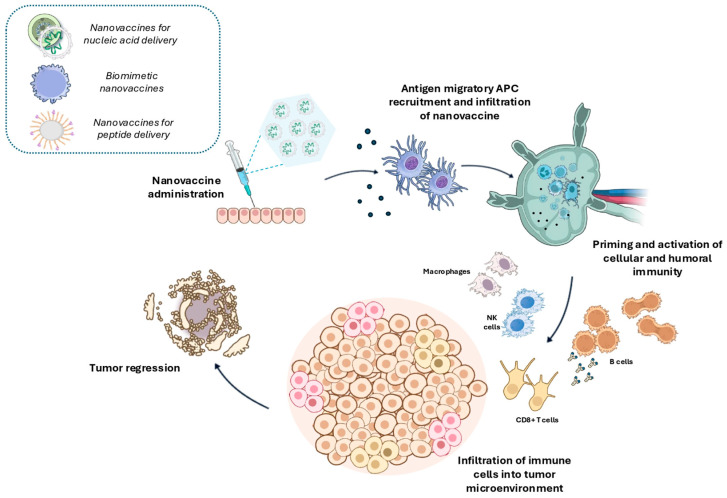
Mechanism of nanovaccine-mediated tumor regression. The schematic illustrates the stages involved, from nanovaccine administration and antigen presentation to immune cell infiltration and tumor regression. The figure was created using NIAID NIH BIOART: source bioart.niaid.nih.gov (accessed on 4 February 2025).

Both antigens and adjuvants play a crucial role in generating an effective antitumor immune response. While antigens themselves are immunogenic, their administration in conjunction with adjuvants significantly improves their effectiveness [224]. Nanovaccines are designed to direct these adjuvants, such as PAMPs, which include CpG, monophosphorylated lipid A, and cyclic GMP, into the lymph nodes, modulating adaptive immune responses and enhancing T-cell and dendritic cell activation. The subcutaneous or intramuscular administration of nanovaccines tends to facilitate their dissemination to peripheral blood, but accumulation in secondary lymphoid organs, such as lymph nodes, is limited, reducing the therapeutic efficacy [225,226]. Recent research has optimized the size of nanoparticles, such as 83 nm polylactic-co-glycolic acid (PLGA), which are shown to be effective in migrating to lymph nodes [227]. This biomaterial has been approved by the Food and Drug Administration (FDA) and the European Medicines Agency (EMA) for clinical use as a drug carrier. Larger nanoparticles have difficulty penetrating lymphatic vesicles, which decreases their therapeutic capacity. On the other hand, innovative strategies include the peritumoral, intratumoral, or intravenous administration of adjuvants, which trap antigens in the tumor microenvironment, facilitating their presentation to dendritic cells and optimizing antitumor immunity [228]. The spleen, recognized for its rapid immune responsiveness, has also been targeted through modifications in the size of nanoparticles and the use of specific ligands, such as albumin, to efficiently target nanovaccines to this organ [229] (Figure 3).

### 6.2. Nanovaccine-Based Systems

#### 6.2.1. Nanovaccines for Nucleic Acid Delivery

Although nucleic acid (DNA and RNA) vaccines have been widely used to treat infectious diseases due to their simplicity, safety, and efficient production, when they are used for cancer treatment, we find problems of low stability, inefficient administration, or limited immunogenicity [230,231]. Intramuscular or subcutaneous administration involves rapid nucleic acid degradation, leading to a limitation in the number of APCs to be transfected. Other strategies, such as electroporation, ultrasound, or gene guns, cause pain and discomfort in patients [232]. That is why strategies such as the use of nanomaterials for cancer vaccination emerge as a solution to these problems, allowing effective and targeted delivery and thereby enhancing immunogenicity. Nucleic acid vaccines based on nanobiomaterials involve the use of virus-like nanoparticles as vaccine carriers [233]. In a study published in 2023, the authors used cGAMP-loaded virus-like particles (VPLs) (cGAMP-VLP) to activate tumor-specific immune responses by stimulating the STING pathway in dendritic cells. This promoted tumor antigen-specific T-cell differentiation and decreased the presence of Treg cells in the tumor microenvironment, enhancing the antitumor response [234]. Likewise, lipid-based nanobiomaterials are among the most widely used non-viral nanoplatforms for nucleic acid delivery [235]. They are often functionalized with specific molecular ligands to ensure the accurate recognition of target cells. In a study conducted by Rudin et al., they analyzed a nanovaccine based on an antisense oligonucleotide (AON) directed against the liposome-formulated c-raf-1 protocogene (LErafAON). The presence of AON in circulation was detected up to 24 h after administration at the highest doses, and in two of the five patients analyzed, the suppression of the c-raf-1 messenger RNA was observed. The liposomal formulation proved to be crucial for AON stability [236]. In recent years, polymeric micelles have emerged as a promising approach for the delivery of nucleic acid vaccines, particularly due to their ability to encapsulate and protect mRNA. These micelles, formed from amphiphilic block copolymers, have the capacity to increase the stability of the mRNA and facilitate its intracellular delivery. By optimizing their composition, polymeric micelles can enhance the biodistribution and targeting of the vaccine while minimizing potential side effects. This system enhances antigen presentation by ensuring that mRNA is efficiently delivered to APCs, thereby improving the overall immune response [237].

In 2020, a functionalized DNA nanoplatform was developed, composed of a rectangular structure formed by the DNA strand of an M12 bacteriophage, to which three extended strands with specific nucleotide sequences had been added to allow the binding of multiple charges. Various peptides, used as antigens, had been conjugated with DNA and bound to the rectangular structure by hybridization so that the peptides remained on the surface. As adjuvants, a double-stranded RNA sequence and a loop-like structure containing three CpG motifs had been incorporated [238]. This nanovaccine was evaluated in a mouse model of melanoma. The animals were treated subcutaneously with the nanovaccine or with control formulations. Its efficacy was assessed by looking at the tumor growth and the survival rate. In addition, bone marrow DCs were incubated with the nanovaccine or controls to analyze the antigenic presentation. The cells treated with the DNA structure and the FITC antigen became known as the physical mixture. The results showed that, in the untreated group, all the mice died within 25 days. The group that received the physical mixture did not show the significant inhibition of tumor growth or increased survival compared to the untreated group. The group that received the vaccine with only the antigen showed mild tumor inhibition, while the group treated with the nanovaccine showed significant tumor regression and maximum therapeutic efficacy, with 60% of the mice surviving for more than 40 days [238].

#### 6.2.2. Biomimetic Nanovaccines

To improve the efficacy and safety of cancer vaccines, recent advances in nanotechnology have led to the development of biomimetic nanovaccines. These nanovaccines are based on the design of nanobiomaterials that mimic natural biological structures, allowing for better interaction with the immune system and a more effective response to cancer [239,240]. The most promising biomimetic nanobiomaterials include nanovesicles derived from tumor cells or immune cells, naturally occurring nanomaterials, membrane fusion materials, and artificial antigen-presenting cells (aAPCs). These innovative systems not only improve antigen delivery but also optimize the activation of immune system cells, increasing the therapeutic capacity of cancer vaccines.

To date, a variety of naturally occurring nanobiomaterials, such as exosomes [241,242], outer membrane vesicles (OMVs) [243,244], and proteins [245,246], have been extensively studied, demonstrating their crucial role in delivering drugs to specific sites in the body. In a study published by Shichuan et al., they developed exosomes derived from tumor cells that were genetically modified to express activated fibroblast protein (FAP)+. These nanovesicles were able to induce the strong immune response of specific CTLs against FAP+ tumor cells and cancer-associated fibroblasts (CAFs), which contributed to the reprogramming of the immunosuppressive tumor microenvironment [247].

On the other hand, biomimetic nanoparticles coated with autologous tumor cell membranes have been developed as a cancer vaccination system [248]. In a study published in 2024, the authors developed a targeted biomimetic system (GMNPs@AMD/RAPA) using the glioblastoma cell membrane (GBM) to improve drug delivery across the blood–brain barrier. The system hierarchically released two therapeutic agents: the CXCR4 antagonist, AMD3100, and the inhibitor of the mTOR pathway, rapamycin (RAPA). They were able to promote the infiltration of CTLs, suppressing the survival, proliferation, and angiogenesis of tumor cells [249].

Likewise, in recent years, nanovaccines derived from cell membranes have been developed based on the fused configuration of DCs with tumor cells. This fusion could induce the processing of whole tumor antigens and costimulatory molecules into cytomembranes. In a paper published by Liu et al., they developed a cancer vaccine using biologically reprogrammed cytoplasmic membranes derived from fused cells (FCs) obtained from DCs and tumor cells. This system works similarly to APCs, directly activating T-cells. In addition, NP@FM contain tumor antigens, which can be recognized by DCs, triggering the DC-mediated activation of T-cells. The combination of these two immunoactivation mechanisms generates a potent antitumor immune response [250].

Finally, within the context of biomimetic nanovaccines, we have aAPCs. They mimic the cellular function of natural APCs to induce a tumor-specific immune response, all without the need to administer specific neoantigens and adjuvants [251]. In a study published by Whang et al., they designed a DC-based nanovaccine called GP@CRTM and evaluated its efficacy in combination with low-dose doxorubicin (DOX) to treat fibrosarcoma. It was a nanoparticle with a PLGA core that encapsulated the STING 2,3-cAMP agonist. The covering was made up of fibrosarcoma cell membranes with high calreticulin expression. The nanovaccine managed to activate the STING pathway in DCs. Furthermore, the administration of DOX at low doses reduced the side effects associated with chemotherapy, increased tumor immunogenicity, and boosted the efficacy of the nanovaccine [252].

#### 6.2.3. Nanovaccines for Peptide Delivery

As we mentioned, cancer vaccines based on antigenic peptides and adjuvants have gained great attention in cancer immunotherapy due to their easy preparation and favorable safety profile [253]. Not only do these vaccines induce antigen-specific antitumor immune responses, but they are also effective in overcoming the immune tolerance that tumors typically develop [254]. In addition, the accelerated advancement of bioinformatic methods has driven the development of personalized vaccines that, by targeting specific neoantigens, generate powerful immune responses tailored to each patient [124]. However, antigenic peptides and neoantigens present significant challenges, such as rapid degradation in the body and low efficiency in delivering to lymph nodes. In this context, nanobiomaterials have emerged as a promising strategy to overcome the limitations associated with the delivery of cancer vaccines [209]. Nanomaterials that can be activated by stimuli are particularly relevant. These, after specific stimulation, can be activated to undergo a structural transition or dissociation to control drug administration and release [255,256,257]. In a study published by Lafuente-Gómez et al., they developed a nanovaccine based on magnetic nanoparticles covalently functionalized with a specific antigen (OVA254-267) and a CpG oligonucleotide, linked by disulfide bonds. Functionalized nanoparticles (MNP-CpG-COVA) demonstrated a significantly superior ability to activate dendritic cells compared to free components. They were also able to induce a robust and sustained antitumor response in specific CTLs against B16-OVA melanoma cells in in vitro experiments [258].

One of the main challenges in the fight against cancer is the immunosuppressive environment generated by tumors, which hinders the effective action of T-cells. In 2023, Liu et al. developed a nanovaccine based on a cholesterol-modified cationic peptide, dubbed DP7-C, that addresses this problem by modifying tumor-induced immunosuppression [259]. The main focus was to create an easy-to-prepare nanometer vaccine that combines small interfering RNAs (siRNAs) in a cocktail and the immune adjuvant CpG ODNs, achieving a synergistic effect in cancer treatment. STAT3 pRNA induces the apoptosis of tumor cells and facilitates the release of specific tumor antigens. In turn, CCR2 pipRNA decreases the inhibition of macrophages and myeloid-derived suppressor cells, while TGF-β piRNA eliminates the immunosuppression of immune cells [260,261]. As for CpG oligodeoxynucleotides, they activate APCs and promote the migration of dendritic cells to the tumor, where they facilitate the cross-presentation of antigens. The vaccine has been tested in mouse models with immunologically active tumors (“hot tumors”) and in tumors resistant to conventional therapy (“cold tumors”). In both cases, it demonstrated the ability to create a favorable environment for effector cells. In addition, its combination with an anti-PD1 antibody significantly increased the number of CD8^+^ and CD4^+^ T-cells. It was also observed to prevent relapses and metastases thanks to its highly specific immune response [259]. Its simplicity and effectiveness position it as a promising tool to combine with other immuno-oncology agents.

### 6.3. Clinical Trials in Nanovaccines for Cancer

Over the past decade, notable advancements have been achieved in the field of cancer nanovaccines, with a growing number of formulations advancing into clinical trials. Table 1 provides an overview of specific clinical trials related to the nanovaccine categories discussed in this review. The information presented in the table has been carefully extracted from ClinicalTrials.gov, a widely recognized and reliable registry for clinical trials. Each trial is listed with its unique NCT identifier, ensuring the accuracy and traceability of the referenced studies. The NANO-GBM study (NCT04881032) is investigating the use of AGuIX^®^ nanoparticles in combination with radiotherapy and temozolomide for treating newly diagnosed glioblastoma. AGuIX^®^ nanoparticles are designed to enhance radiotherapy by amplifying radiation damage specifically in tumor tissues. Phase I determined the recommended dose for phase II (100 mg/kg) and assessed the safety and biodistribution of AGuIX^®^ in glioblastoma patients. Phase II is a multicenter, randomized trial comparing two groups: one receiving AGuIX^®^ combined with standard radiochemotherapy and another receiving radiochemotherapy alone. The early results show promising safety and efficacy, with the final data expected in 2026 [262]. The clinical trial NCT00609791 is a phase II study focused on evaluating the use of a paclitaxel albumin-stabilized nanoparticle formulation (nab-paclitaxel) in treating patients of different ages with metastatic breast cancer. The patients receive nab-paclitaxel intravenously once weekly on days 1, 8, and 15 in a 28-day cycle, which is repeated as long as there is no disease progression or unacceptable toxicity. Although the trial is still active (not recruiting), the preliminary findings indicate age-related differences in drug clearance and toxicity levels. The drug is generally effective, with outcomes such as response rates and the time to progression being monitored. Factors like age and baseline health significantly influence the need for dose reductions and toxicity management [263]. In another study (NCT03739931), researchers investigated the use of mRNA-2752, which is encapsulated in lipid nanoparticles, for intratumoral injection in patients with advanced malignancies. The mRNA-2752 encodes human OX40L, IL-23, and IL-36γ, which are designed to enhance the immune response and potentially improve the efficacy of immune checkpoint blockade therapy. The combination therapy led to increased levels of pro-inflammatory cytokines and activated immune cells, particularly CD8^+^ T-cells, which are essential for antitumor immunity. Objective responses, such as partial responses (PR) and complete responses (iCR), were observed in patients with immune-refractory tumors, including TNBC and melanoma. Additionally, no severe adverse events (grade 4 or 5) were reported, confirming the treatment’s safety profile [264]. A phase I clinical trial (NCT02716012) involves the use of MTL-CEBPA, a novel therapeutic that utilizes liposomal nanoparticles for drug delivery in patients with advanced liver cancer, specifically hepatocellular carcinoma (HCC). The results indicated that MTL-CEBPA had an acceptable safety profile, with no dose-limiting toxicities observed. In terms of efficacy, the trial demonstrated promising results: one patient achieved a partial response lasting over two years, and 50% of the patients had a stable disease [265]. In conclusion, the clinical trials of nanovaccines for cancer have demonstrated a promising potential in enhancing the effectiveness of cancer immunotherapy. These trials explore the use of various nanomaterial-based platforms, such as lipid nanoparticles, dendritic cell mimicking systems, and virus-like particles, to improve the delivery and presentation of tumor antigens. As these therapies progress through clinical stages, they provide hope for more personalized, effective treatments that could complement existing cancer therapies and potentially lead to better patient outcomes. However, continued research and larger clinical trials are needed to fully establish their safety, efficacy, and long-term impact on cancer treatment.

## 7. Conclusions and Future Directions

In the pursuit of effective immunotherapeutic vaccines for cancer treatment, a nuanced understanding of the immune system’s complexities is essential. Cancer is a dynamic disease characterized by continuous mutations and a myriad of mechanisms that allow it to evade immune detection, including immunoediting and the establishment of an immunosuppressive tumor microenvironment. These factors complicate the development of viable vaccine therapies; nonetheless, advancements in this field mark a transformative step forward in oncology. Therapeutic vaccines represent a groundbreaking approach that engages the immune system in actively targeting and eliminating malignant cells. Cell-based vaccines, particularly those utilizing dendritic cells, effectively present tumor antigens and activate T-cells, creating a robust immune response. The efficacy of these vaccines relies heavily on enhancing antigen presentation by APCs, a crucial factor for the activation of both CD4^+^ and CD8^+^ T-cell responses. As we explore peptide and nucleic acid vaccines, it is evident that the strategic selection of strong and specific antigens, including neoantigens derived from tumor-specific mutations, is vital. The integration of novel delivery systems, such as lipid nanoparticles and viral vectors, has further improved the immunogenicity and safety profile of these vaccines, providing promising avenues for future research. Moreover, mRNA vaccines have demonstrated the potential for rapid production and the capacity to induce both humoral and cellular immune responses, making them a highly versatile tool in cancer immunotherapy. Personalized cancer vaccines, designed to target the unique antigenic profiles of individual tumors, could significantly improve treatment responses and mitigate the risk of recurrence. Ongoing advancements in genomic technologies, such as whole-exome sequencing and advanced bioinformatics, stand to play a critical role in identifying the most effective neoantigens for vaccine development.

Nevertheless, the focus on nanovaccines, as a central point of innovation, highlights their significant potential in the landscape of cancer immunotherapy. By leveraging advancements in nanotechnology, these vaccines address the critical challenges faced by traditional vaccine formulations, such as rapid antigen degradation, low stability, and inadequate immunogenicity. Nanovaccines enhance the targeted delivery of antigens to immune cells, thereby facilitating the more effective activation of the immune response. Moreover, their ability to co-deliver multiple tumor antigens and immune-stimulating agents offers a promising avenue to counter tumor heterogeneity while minimizing off-target effects. In the future, ongoing research must focus on optimizing nanovaccine formulations to improve their efficacy, safety, and mechanistic understanding. Strategies that combine nanovaccines with immunomodulators, such as immune checkpoint inhibitors, could further potentiate their therapeutic effects. The integration of personalized elements into nanovaccine design, based on individual tumor profiles and unique neoantigens, is a crucial area that requires additional investigation to maximize the specificity and effectiveness of treatments. Furthermore, the adoption of biomimetic nanovaccines, which mimic natural biological structures, holds promise for enhancing interaction with the immune system and improving therapeutic outcomes. By utilizing these advanced strategies, researchers can increase the chances of overcoming the immunosuppressive mechanisms employed by tumors, thereby fostering more robust antitumor immunity. Despite the significant potential of nanovaccines, further research is needed to address challenges associated with scalable manufacturing, ensuring stability during storage, and navigating regulatory pathways for personalized therapies. Overcoming these obstacles will be crucial for maximizing the impact of nanovaccines on cancer treatment.

In summary, although significant challenges remain in the landscape of cancer immunotherapy, the trajectory of research and innovation is encouraging. Continued efforts in refining immunotherapeutic vaccines, expanding their application across various cancer types, and striving for personalized treatment strategies will be essential for enhancing their effectiveness. Nanovaccines exemplify the forefront of cancer immunotherapy, harnessing innovative technology to address existing limitations and provide enhanced therapeutic responses. As research continues to refine these strategies and translate findings from bench to bedside, nanovaccines are poised to play a pivotal role in shaping the future landscape of cancer treatment. By fostering interdisciplinary collaboration, investing in technological advancements, and prioritizing patient-centered approaches, we can envision a future where nanovaccines and immunotherapeutic strategies collectively contribute to more effective and personalized cancer therapies, ultimately offering renewed hope for patients worldwide.

## Figures and Tables

**Table 1:** Clinical trials of nanovaccines for cancer treatment. Source: https://clinicaltrials.gov/ (accessed on 15 December 2024). NP—not published.

NCT Number	Status	Start/Completion Date	Stage	Title	References
NCT05264974	Suspended	-	Phase I	Novel RNA-nanoparticle Vaccine for the Treatment of Early Melanoma Recurrence Following Adjuvant Anti-PD-1 Antibody Therapy	NP
NCT04645147	Active, not recruiting	2022-03/present	Phase I	Safety and Immunogenicity of an Epstein-Barr Virus (EBV) gp350-Ferritin Nanoparticle Vaccine in Healthy Adults With or Without EBV Infection	NP
NCT03120832	Completed	2016-12/2018-12	Phase I	Phase 1 Trial of PAN-301-1 (SNS-301) in Cancer Patients	NP
NCT03606967	Recruiting	2021-04/present	Phase II	Testing the Addition of an Individualized Vaccine to Durvalumab and Tremelimumab and Chemotherapy in Patients With Metastatic Triple Negative Breast Cancer	NP
NCT05456022	Unknown status	2022-07/-	Phase II	Therapeutic Efficacy of Quercetin Versus Its Encapsulated Nanoparticle on Tongue Squamous Cell Carcinoma Cell Line	NP
NCT04881032	Active, not recruiting	2022-03/present	Phase I Phase II	AGuIX Nanoparticles with Radiotherapy Plus Concomitant Temozolomide in the Treatment of Newly Diagnosed Glioblastoma (NANO-GBM)	[262]
NCT03410030	Completed	2017-12/2022-01	Phase I Phase II	Trial of Ascorbic Acid (AA) + Nanoparticle Paclitaxel Protein Bound + Cisplatin + Gemcitabine (AA NABPLAGEM) (AA NABPLAGEM)	NP
NCT05000801	Recruiting	2021-07/present	Not Applicable	Clinical Study of DC-AML Cells in the Treatment of Acute Myeloid Leukemia	NP
NCT00609791	Active, not recruiting	2008-02/present	Phase II	Paclitaxel Albumin-Stabilized Nanoparticle Formulation in Treating Patients of Different Ages With Metastatic Breast Cancer	[263]
NCT03323398	Terminated	2017-08/2021-08	Phase I Phase II	Dose Escalation and Efficacy Study of mRNA-2416 for Intratumoral Injection Alone and in Combination With Durvalumab for Participants With Advanced Malignancies	NP
NCT05968326	Recruiting	2023-10/present	Phase II	A Study of the Efficacy and Safety of Adjuvant Autogene Cevumeran Plus Atezolizumab and mFOLFIRINOX Versus mFOLFIRINOX Alone in Participants With Resected PDAC (IMCODE003)	NP
NCT02149225	Completed	2014-10/2018-06	Phase I	GAPVAC Phase I Trial in Newly Diagnosed Glioblastoma Patients	NP
NCT03739931	Active, not recruiting	2018-11/present	Phase I	Dose Escalation Study of mRNA-2752 for Intratumoral Injection to Participants in Advanced Malignancies	[264]
NCT05533697	Recruiting	2022-08/present	Phase I Phase II	Study of mRNA-4359 Administered Alone and in Combination With Immune Checkpoint Blockade in Participants With Advanced Solid Tumors	NP
NCT02716012	Active, not recruiting	2016-03/present	Phase I	First-in-Human Safety, Tolerability and Antitumor Activity Study of MTL-CEBPA in Patients With Advanced Liver Cancer (OUTREACH)	[265]
NCT05631886	Recruiting	2023-07/present	Phase I	Combination of CAR-DC Vaccine and ICIs in Malignant Tumors	NP
NCT02975882	Active, not recruiting	2017-08/present	Phase I	Nanoparticle Albumin-Bound Rapamycin, Temozolomide, and Irinotecan Hydrochloride in Treating Pediatric Patients With Recurrent or Refractory Solid Tumors	NP
NCT03313778	Recruiting	2017-08/present	Phase I	Safety, Tolerability, and Immunogenicity of mRNA-4157 Alone and in Combination in Participants With Solid Tumors (KEYNOTE-603)	NP
NCT03897881	Recruiting	2019-07/present	Phase II	An Efficacy Study of Adjuvant Treatment With the Personalized Cancer Vaccine mRNA-4157 and Pembrolizumab in Participants With High-Risk Melanoma (KEYNOTE-942)	NP
NCT05062980	Active, not recruiting	2022-03/present	Phase I Phase II	Quaratusugene Ozeplasmid (Reqorsa) in Combination with Pembrolizumab in Previously Treated Non-Small Lung Cancer (Acclaim-2)	NP
NCT01847326	Completed	2013-03/2024-01	Phase I	Paclitaxel Albumin-Stabilized Nanoparticle Formulation and Carboplatin Followed By Chemoradiation in Treating Patients With Recurrent Head and Neck Cancer	[266]
NCT06048367	Recruiting	2022-10/present	Phase I	Carbon Nanoparticle-Loaded Iron [CNSI-Fe(II)] in the Treatment of Advanced Solid Tumor (CNSI-Fe(II))	[267,268,269]
NCT01435720	Unknown status	2011-09/-	Phase I Phase II	Safety and Tolerability Study of SNS01-T in Relapsed or Refractory B Cell Malignancies (Multiple Myeloma, B Cell Lymphoma, or Plasma Cell Leukemia (PCL)	NP
NCT01676259	Unknown status	2018-03/-	Phase II	A Phase 2 Study of siG12D LODER in Combination With Chemotherapy in Patients With Locally Advanced Pancreatic Cancer (PROTACT)	NP
NCT00436410	Completed	2006-12/2009-08	Early Phase I	Tumor Necrosis Factor in Patients Undergoing Surgery for Primary Cancer or Metastatic Cancer	NP
NCT03020017	Completed	2017-05/2020-08	Early Phase I	NU-0129 in Treating Patients With Recurrent Glioblastoma or Gliosarcoma Undergoing Surgery	NP
NCT01159288	Completed	2010-05/2015-12	Phase II	Trial of a Vaccination With Tumor Antigen-loaded Dendritic Cell-derived Exosomes (CSET 1437)	NP
NCT00651703	Completed	2008-04/2009-12	Phase II	Safety and Immunogenicity of CYT004-MelQbG10 Vaccine With and Without Adjuvant in Advanced Stage Melanoma Patients	NP
NCT03206073	Completed	2017-12/2022-06	Phase I Phase II	A Phase I/II Study of Pexa-Vec Oncolytic Virus in Combination With Immune Checkpoint Inhibition in Refractory Colorectal Cancer	[270,271,272]
